# Prostate Cancer and Its Mimics—A Pictorial Review

**DOI:** 10.3390/cancers15143682

**Published:** 2023-07-19

**Authors:** Anna Żurowska, Rafał Pęksa, Michał Bieńkowski, Katarzyna Skrobisz, Marek Sowa, Marcin Matuszewski, Wojciech Biernat, Edyta Szurowska

**Affiliations:** 1 Second Department of Radiology, Medical University of Gdańsk, 80-214 Gdańsk, Poland; edyta.szurowska@gumed.edu.pl; 2 Department of Pathomorphology, Medical University of Gdańsk, 80-214 Gdańsk, Poland; rafalpeksa@gumed.edu.pl (R.P.); michal.bienkowski@gmail.com (M.B.); wojciech.biernat@gumed.edu.pl (W.B.); 3 Department of Radiology, Medical University of Gdańsk, 80-214 Gdańsk, Poland; kskrobisz@gumed.edu.pl; 4 Department of Urology, Medical University of Gdańsk, 80-214 Gdańsk, Poland; mareksowa88@gmail.com (M.S.); marcin.matuszewski@gumed.edu.pl (M.M.)

**Keywords:** prostate cancer, magnetic resonance imaging (MRI), mpMRI, pitfalls, differential diagnosis

## Abstract

**Simple Summary:**

This review focuses on the potential challenges encountered in the interpretation of multiparametric prostate MRI (mpMRI). While mpMRI is accurate in the diagnosis of prostate cancer, false positive and false negative results can occur. The review discusses anatomical structures and benign conditions that may mimic prostate cancer, including prostatitis, ectopic and stromal benign prostate hyperplasia nodules, post-biopsy hemorrhage, and abscesses. The article provides suggestions on avoiding these pitfalls and emphasizes the importance of image quality in achieving accurate interpretations. Radiologists need to be aware of these pitfalls to enhance diagnostic accuracy in the increasingly prevalent use of mpMRI for prostate evaluation.

**Abstract:**

Background: Multiparametric prostate MRI (mpMRI) is gaining wider recommendations for diagnosing and following up on prostate cancer. However, despite the high accuracy of mpMRI, false positive and false negative results are reported. Some of these may be related to normal anatomic structures, benign lesions that may mimic cancer, or poor-quality images that hamper interpretation. The aim of this review is to discuss common potential pitfalls in the interpretation of mpMRI. Methods: mpMRI of the prostates was performed on 3T MRI scanners (Philips Achieva or Siemens Magnetom Vida) according to European Society of Urogenital Radiology (ESUR) guidelines and technical requirements. Results: This pictorial review discusses normal anatomical structures such as the anterior fibromuscular stroma, periprostatic venous plexus, central zone, and benign conditions such as benign prostate hyperplasia (BPH), post-biopsy hemorrhage, prostatitis, and abscess that may imitate prostate cancer, as well as the appearance of prostate cancer occurring in these locations. Furthermore, suggestions on how to avoid these pitfalls are provided, and the impact of image quality is also discussed. Conclusions: In an era of accelerating prostate mpMRI and high demand for high-quality interpretation of the scans, radiologists should be aware of these potential pitfalls to improve their diagnostic accuracy.

## 1. Introduction

Prostate cancer (PCa) is one of the most common malignant tumors in men, with an incidence of over 1.4 million worldwide [1]. Multiparametric prostate MRI (mpMRI) is commonly used in the detection, staging, active surveillance, and follow-up after treatment of PCa. This technique combines excellent morphological images (T1, T2-weighted) with functional imaging: diffusion-weighted imaging (DWI) and dynamic contrast enhanced imaging (DCE). Recent trials have also revealed the great potential of mpMRI for performing pre-biopsy diagnosis [2,3,4,5,6]. As a result, the latest 2023 European Association of Urology (EAU) guidelines recommend prostate MRI as one of the tools for biopsy indication in asymptomatic men with PSA 3–10 ng/mL and normal DRE [7].

The accuracy of prostate MRI is influenced by the experience of radiologists as well as the diagnostic quality of the imaging [8]. Normal anatomical structures and benign findings may sometimes have a similar appearance to PCa on MRI and constitute potential pitfalls in the interpretation that lead to false-positive results. Nevertheless, some of them may be easily identified and recognized. In contrast, others may show overlapping appearance features with PCa, which makes them difficult, or sometimes even impossible, to distinguish from cancer only based on MRI images. This phenomenon may explain a considerable amount of benign findings found in histopathology, even in lesions of assessment categories PI-RADS (Prostate Imaging–Reporting and Data System) 4 and 5 [9].

According to McNeal [10], four anatomical zones are distinguished in the prostate gland: the peripheral zone (PZ), transition zone (TZ), central zone (CZ), and anterior fibromuscular stroma (AFMS).

Around 70–75% of prostate cancers are located in the peripheral zone, 20–30% in the transition zone, and a small percentage (2.5–7%) in the central zone [11,12,13]. AFMS does not constitute the origin of PCa but may be invaded by transition zone cancers [11].

On MRI images, typical prostate cancer presents as a focal lesion of low intensity on T2-weighted images, which restricts diffusion (present as a high signal on DWI and low on ADC maps) and shows focal, earlier, or contemporaneous enhancement with adjacent normal prostatic tissues after intravenous contrast injection [11].

Potential pitfalls related to the anatomic structures, benign lesions, and poor quality of images will be discussed in this review. The summary of prostate cancer mimics in relation to anatomic location is presented in Table 1.

Multiparametric MRIs of the prostates were performed on 3T MRI scanners (Philips Achieva 3.0 T Tx, Best, Netherlands or Siemens 3T Magnetom Vida, Erlangen, Germany) according to European Society of Urogenital Radiology (ESUR) guidelines and technical requirements [18] and PI-RADS ver. 2.1 [11]. Examinations were performed with superficial phase array coils (a 32-channel cardiac coil on Philips or a Contour 24 coil on Siemens). The prostate mpMRI protocol consisted of high-resolution T2-weighted images in three planes, T1-weighted images, diffusion-weighted imaging (DWI), which consisted of 6 b values (0–2000 s/mm^2^) on Philips or 4 b values (50–2000 s/mm^2^) on Siemens, and dynamic contrast-enhanced (DCE) imaging. Throughout the article, lesions were assigned PI-RADS assessment categories according to PI-RADS version 2.1 [11].

## 2. Anterior Fibromuscular Stroma

The anterior fibromuscular stroma is the most anterior part of the prostate. It is located anteriorly to the transitional zone in the midline and paramidline between the two lobes. It extends from the apex to the base of the prostate. AFMS consists of muscle cells and dense connective tissue, without glandular tissue. It merges with the prostate capsule but, in 89% of cases, is not covered by the capsule and, as a result, is the most outer layer of the anterior prostate [19].

**Figure 1:**
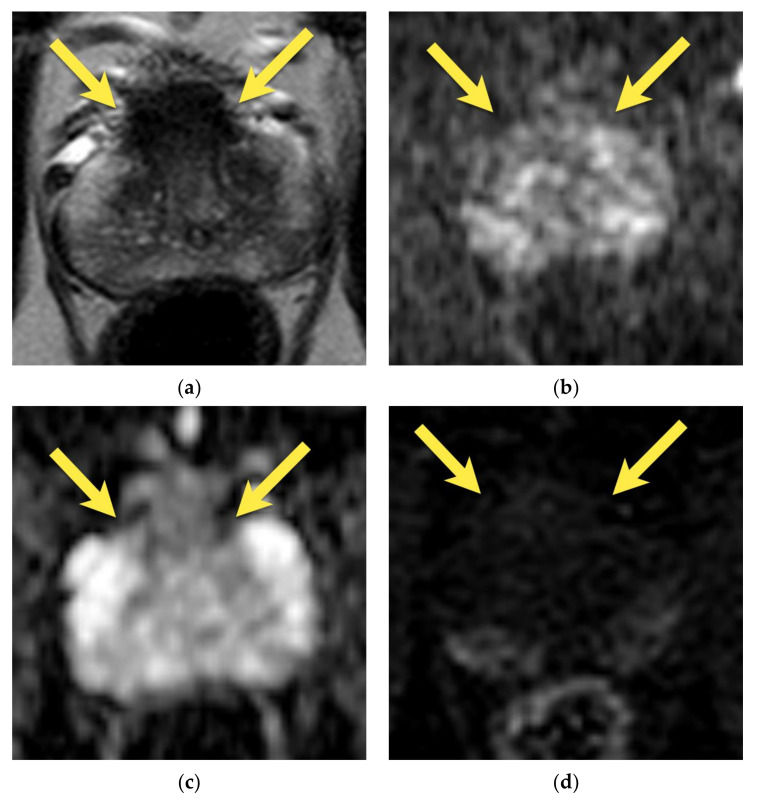

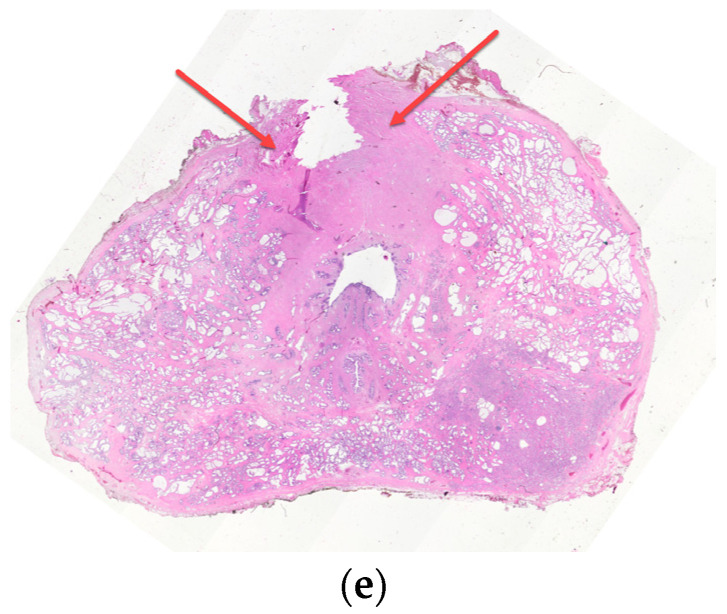
Hypertrophied anterior fibromuscular stroma (normal anatomic structure—PI-RADS 1): axial (**a**) T2-w image presents hypertrophied AFMS with low signal intensity (arrows); with no diffusion restriction (**b**) DWI b 2000 image does not show increased signal intensity (arrows); (**c**) on ADC map it presents as low signal intensity due to T2-dark through effect (arrows); (**d**) and no enhancement on DCE (arrows); (**e**) whole mount histopathology after radical prostatectomy confirms the diagnosis depicting anterior fibromuscular stroma in the anterior part of the prostate (arrows).

**Figure 2:**
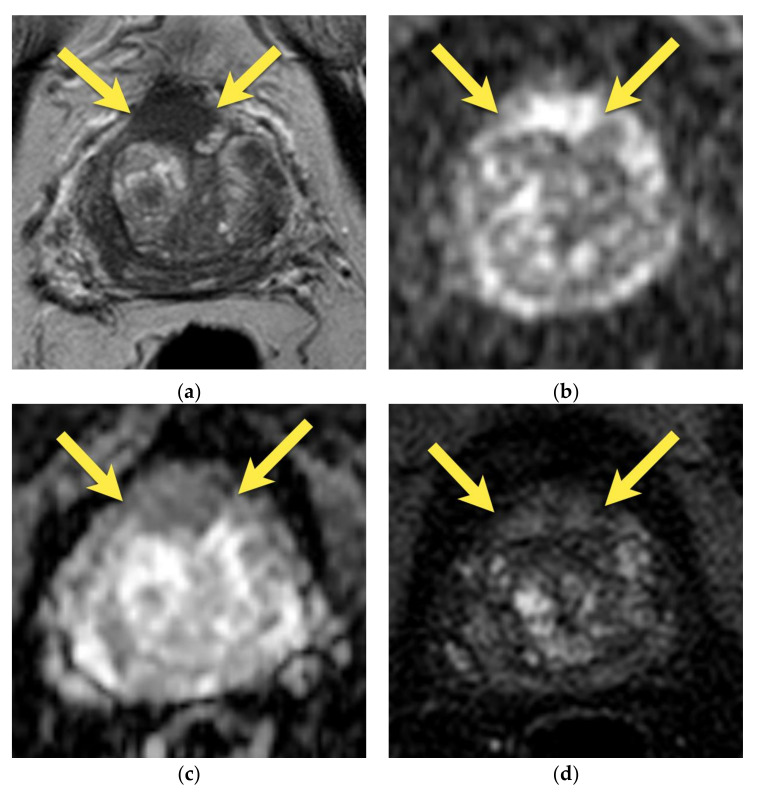

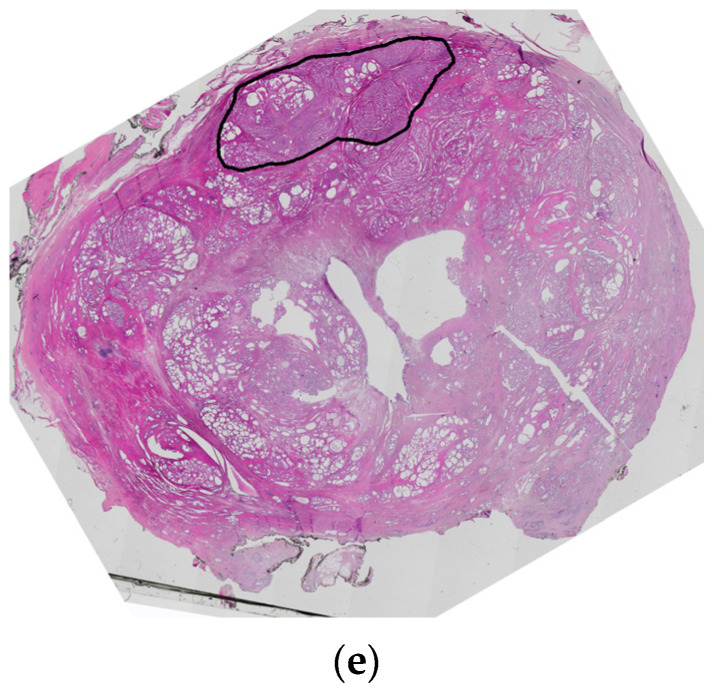
Anterior prostate cancer invading AFMS (PI-RADS 5): axial (**a**) T2-w image presents a lenticular lesion in place of AFMS with low intensity signal of “erased charcoal” appearance (arrows); with diffusion restriction (**b**) high signal on DWI b 2000 image (arrows); (**c**) and low signal on ADC map (arrows); (**d**) early enhancement on DCE (arrows); (**e**) whole mount histopathology after radical prostatectomy reveals prostate cancer GS 4 + 3 in the anterior part of the prostate invading AFMS (outlined with a black continuous line).

On MRI, AFMS appears homogenously hypointense on T2-weighted images due to its histologic composition (comparably with the muscles). Usually, it is slightly more hypointense than prostate cancer, which presents the “erased charcoal” signal pattern. However, when AFMS is hypertrophied, especially asymmetrically, it may mimic PCa and have a similar appearance on T2-weighted images [11,20]. On the contrary to cancer, AFMS never restricts diffusion—it may be hypointense on the ADC map due to the T2-dark through effect but is never hyperintense on DWI and does not show early enhancement on DCE [21] (Figure 1). These two parameters are crucial in distinguishing AFMS hypertrophy from cancer, which usually presents diffusion restriction and may show early focal enhancement on DCE (Figure 2).

Additionally, examining AFMS in three planes in T2-w images (axial, sagittal, and coronal) is highly recommended to confirm the continuity with benign tissue [14].

About 20% of all prostate cancers are located anteriorly. An amount of 53.3% of PCa situated in the transition zone occupies the anterior one-third of the TZ and may abut and infiltrate AFMS. A total of 87.7% of all TZ PCa are located in the anterior two-thirds of the TZ [21].

## 3. Periprostatic Venous Plexus

The periprostatic veins that form the periprostatic venous plexus are located on both lateral sides of the prostate gland. They connect with the venous plexus anterior to the prostate and drain into the internal iliac veins [15,22]. The size of the periprostatic venous plexus varies among individuals, with a tendency to decrease in size with age [23] and be more prominent in men with prostatitis [15].

The periprostatic venous plexus runs near the anatomical capsule of the gland and may eventually be embedded within it on the lateral side within some distance [22]. On T2-w images, veins usually appear as bright, tubular structures; nevertheless, their signal intensity may be variable due to turbulent flow or different blood flow velocities [24].

**Figure 3:**
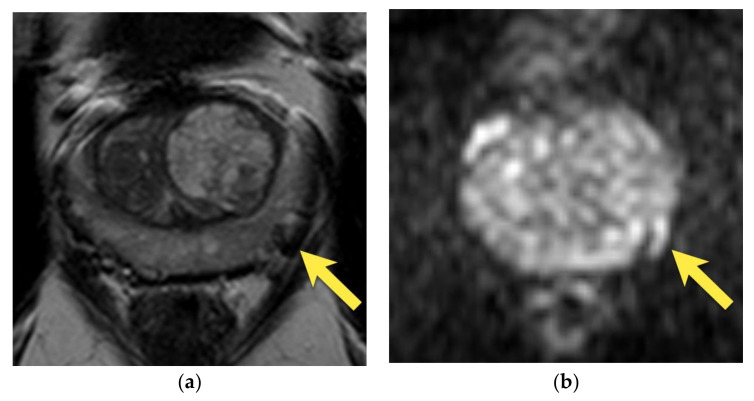

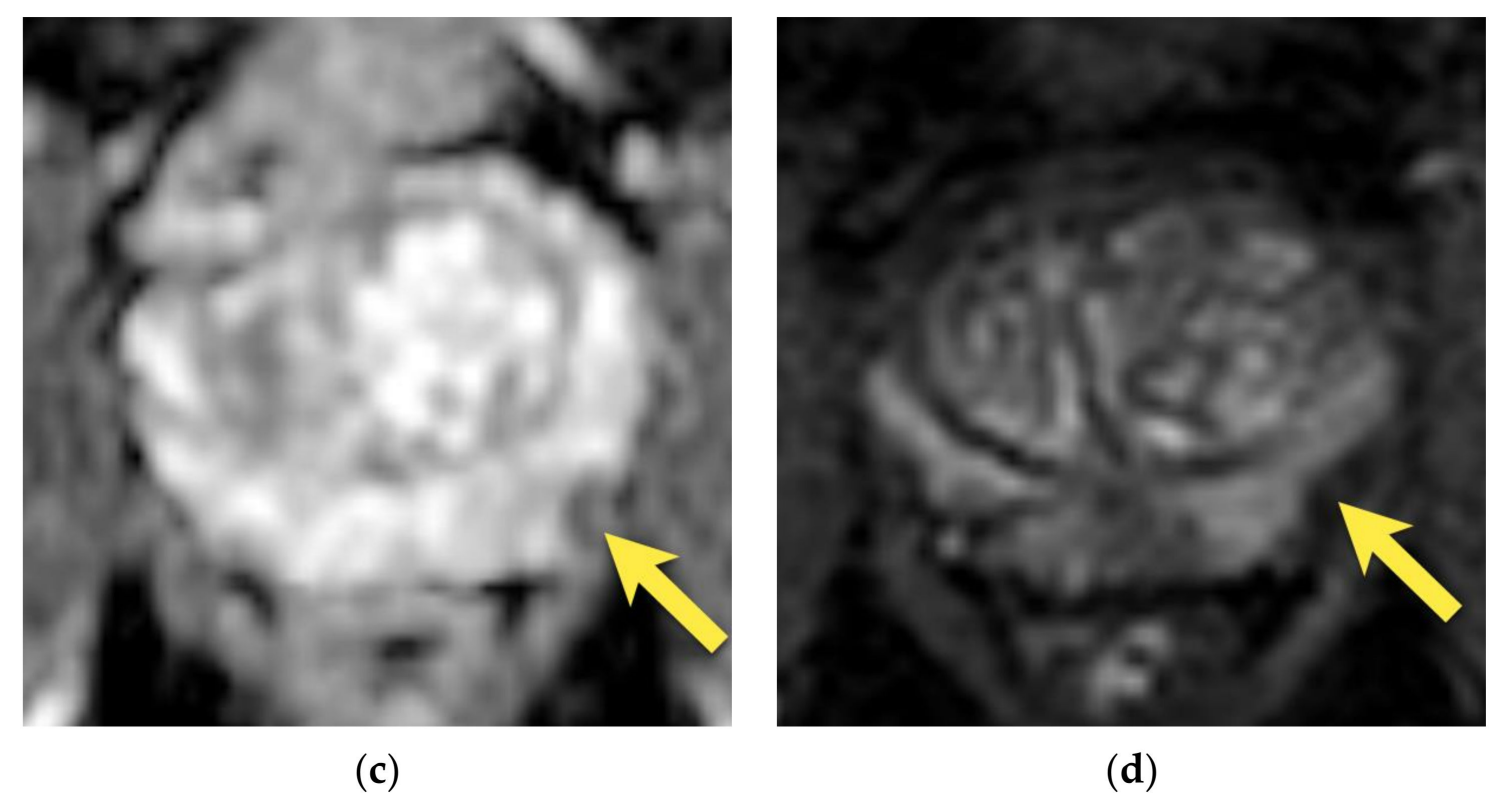
Periprostatic venous plexus in the left postero-lateral side of the prostate, presenting on axial images as an oval structure with: (**a**) intermediate to low signal intensity on T2-w image on the left postero-lateral side of prostate, due to turbulent flow (arrow); moderate diffusion restriction; (**b**) increased signal on DWI b 2000 image (arrow); (**c**) decreased signal on ADC map (arrow); (**d**) but with no early focal enhancement (arrow).

Eventually, periprostatic veins may present as tubular or round structures (if axially imaged) that are hypointense on T2-w images with enhancement on DCE and sometimes mildly hyperintense on DWI owing to a slow velocity of blood flow and therefore mimic prostate carcinoma [14]. However, careful examination of these structures in all three planes should reveal their continuity with the rest of the vessels (Figure 3).

## 4. Central Zone

The central zone has a pyramidal or conical shape and is located bilaterally in the base of the prostate, surrounding the ejaculatory ducts and narrowing towards the level of verumontanum [17]. It contains about 20% of glandular tissue [11], but only 2.5–7% [12,13] of prostate cancers are reported to arise from this zone. However, those cancers tend to be more aggressive due to the early involvement of seminal vesicles and extraprostatic extension [12,13,15,17]. On T2-weighted images, the central zone looks like homogenous, symmetric, oval areas of low signal intensity located at the base of the prostate.

**Figure 4:**
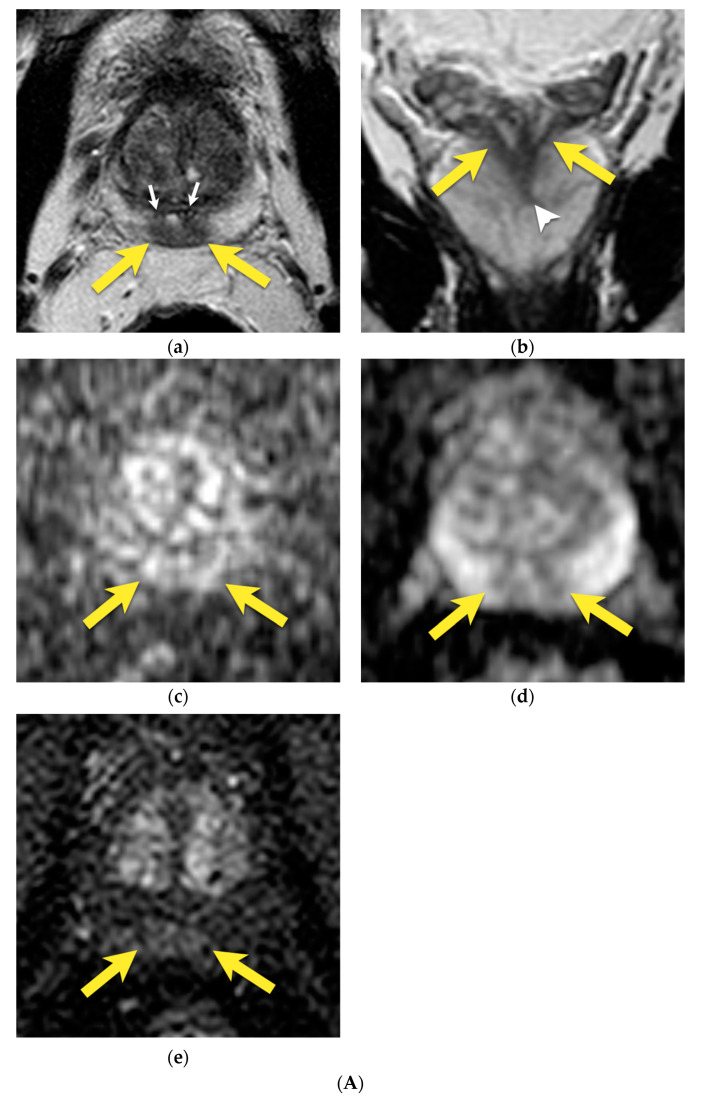

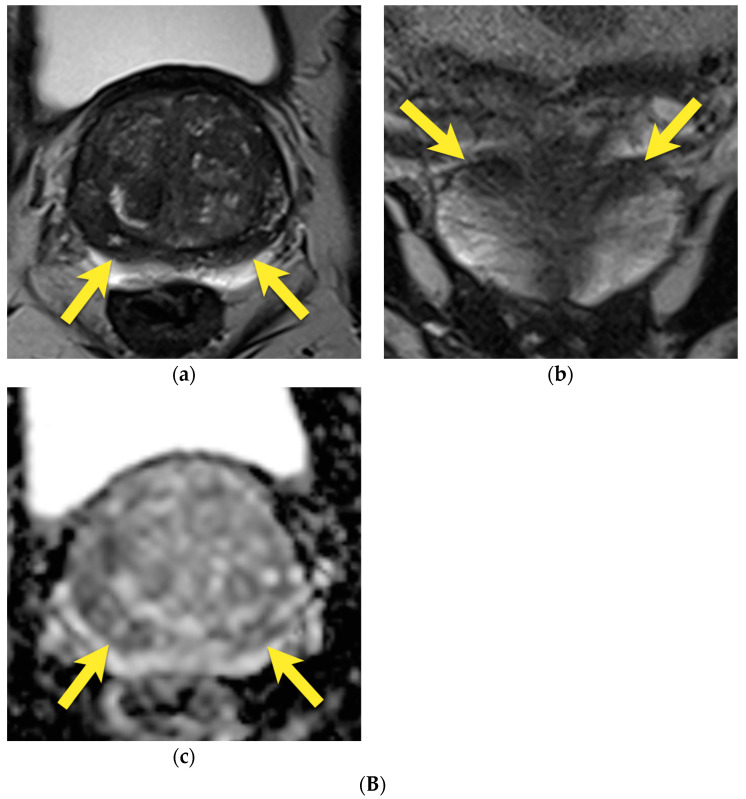
(**A**) Normal central zone: (**a**) axial T2-w image shows homogenously hypointense, symmetric area at the base of the prostate (yellow arrows), surrounding ejaculatory ducts (white arrows); (**b**) coronal T2-w image shows homogenously hypointense, symmetric area in conical shape extending from the base of the prostate (yellow arrows) to verumontanum (white arrowhead); (**c**) with mildly increased signal on DWI b 2000 image (yellow arrows); (**d**) and mildly decrease on ADC map (yellow arrows); (**e**) and diffuse little enhancement of progressive type (yellow arrows). (**B**) Patient with enlarged TZ due to BPH. The central zone in this patient is compressed between enlarged TZ and normal PZ and displaced towards the base of the prostate—the so-called “moustache sign”: (**a**) axial and (**b**) coronal T2-w images show homogenously hypointense, symmetric area at the base of the prostate (arrows); (**c**) with mildly decreased signal on ADC map (arrows).

On the DWI/ADC sequence, it may show diffusion restriction and usually no or weak enhancement on DCE [14], usually with progressive (type 1) or plateau (type 2) enhancement curves [25] (Figure 4A). These malignant-looking morphological and functional features may be mistaken for bilateral prostate cancer.

As the transitional zone becomes enlarged with benign prostatic hyperplasia (BPH), the central zone is compressed and displaced towards the base of the prostate [15,16]. Its shape resembles a mustache in the axial and coronal planes at the base of the prostate—the so-called “mustache sign” [14] (Figure 4B).

The main feature that suggests benignity is the symmetry of the findings—their oval, symmetric, well-defined shape, with a homogenously low-intensity signal on T2-weighted images. The coronal plane may be especially useful to show the normal anatomy of CZ.

**Figure 5:**
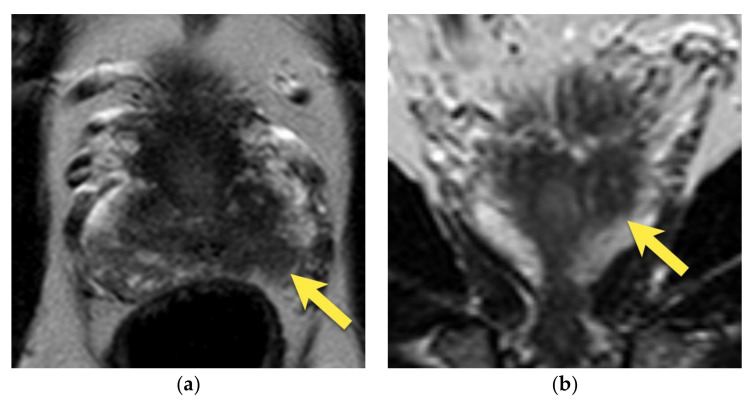

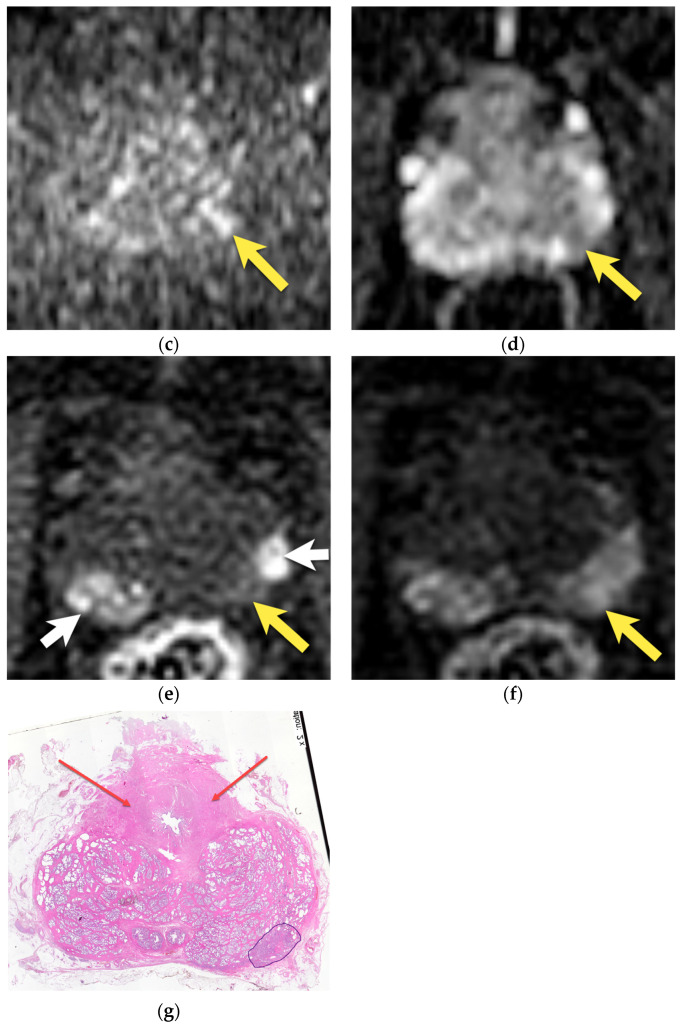
Prostate cancer in central zone: T2-weighted images show the focal asymmetric area of low signal intensity adjacent to the left central zone in axial (**a**) and coronal (**b**) planes (arrow), with diffusion restriction on (**c**) DWI b 2000 image (arrow) and (**d**) ADC map (arrow), and early focal enhancement (**f**). The lesion was assessed as PI-RADS 4; (**e**) native T1-w fat-saturated images showing postbiopsy hemorrhage in the PZ (white arrows); (**f**) focal enhancement of the lesion adjacent to the left CZ (yellow arrow); (**g**) whole mount histopathology after radical prostatectomy revealing prostate cancer GS 4 + 3 in this area (outlined with continuous line). Additionally, on the images, the hypertrophied AFMS is visible in the anterior part of the prostate with low signal intensity on axial T2-w image (**a**), no diffusion restriction (**c**,**d**), and no enhancement (**f**), confirmed at whole mount histopathology after radical prostatectomy (red arrows) (**g**).

On the contrary, prostate cancers tend to be more heterogeneous on T2-weighted images with ill-defined margins, which results in asymmetric irregularities in the central zone in T2-w images. If such a lesion additionally presents marked diffusion restriction and focal, early enhancement (more frequently, it is associated with a type 3 (washout) enhancement curve), it suggests a suspicious lesion [14,21] (Figure 5).

Irregular hypertrophy of the transitional zone may lead to the asymmetric displacement of the central zone and present as a pseudolesion [16], making a correct diagnosis even more difficult.

## 5. Hypointense Area in the Median Posterior Middle Gland or at the Base

Often, a focal area that is hypointense in T2-weighted images may be observed in the midline of the posterior middle gland or at the base of the prostate. It is usually symmetric and wedged in shape in T2-weighted images and may show moderate diffusion restriction and focal, slight contrast enhancement, thus mimicking cancer.

These areas may, however, correspond to the apex of the central zone (compressed between the transition zone and peripheral zone). As the central zone extends into the middle third of the prostate, its shape resembles a teardrop in the coronal plane—so called the “teardrop sign” [14,15]. In order to interpret this finding, it is necessary to visualize the continuity of hypointense areas of the median posterior middle gland with the central zone at the base of the prostate in the coronal plane and assess its symmetry and homogeneity.

**Figure 6:**
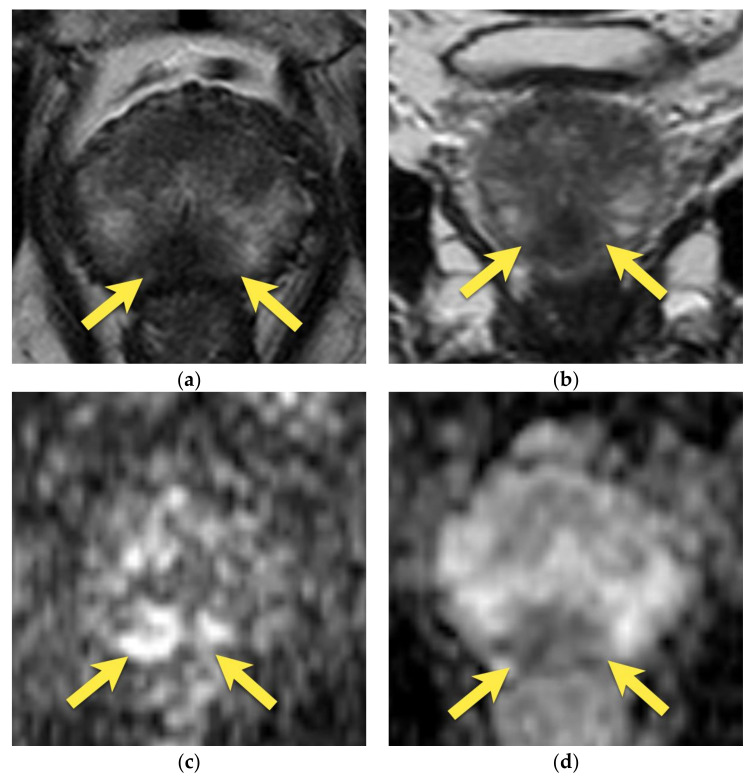

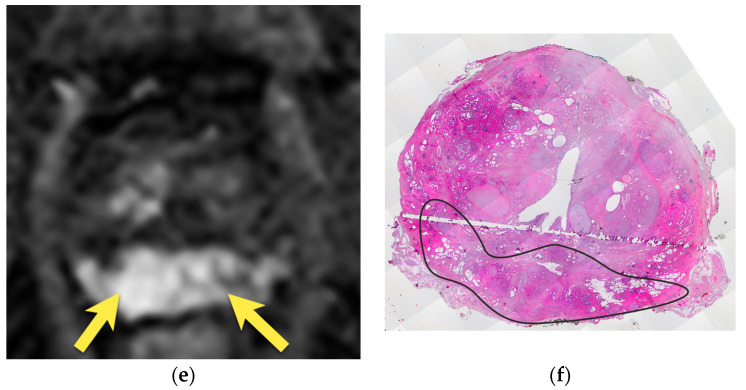
Prostate cancer in the median posterior gland in a patient with PSA 21.36 ng/mL (**a**) axial and (**b**) coronal T2-weighted images showing a focal, ill-defined area of low signal intensity located in the median posterior area at the apex and middle third of the prostate (arrows), with marked diffusion restriction on (**c**) DWI b 2000 image (arrows) and (**d**) ADC map (arrows); and (**e**) early enhancement (arrows). The lesion was assessed as PI-RADS 5. (**f**) After radical prostatectomy, PCa GS 5 + 4 was diagnosed in this location (area outlined with black continuous line).

**Figure 7:**
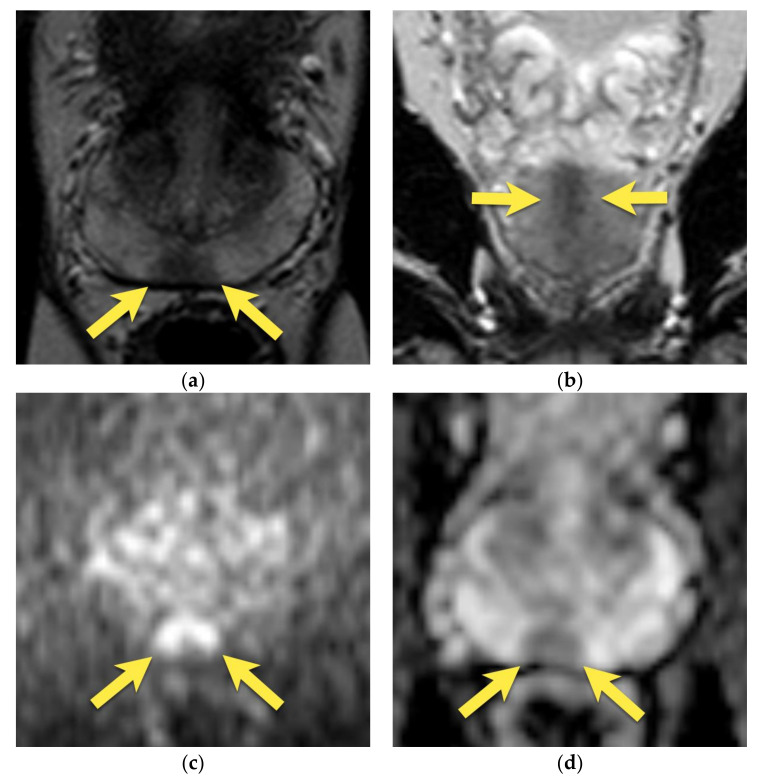

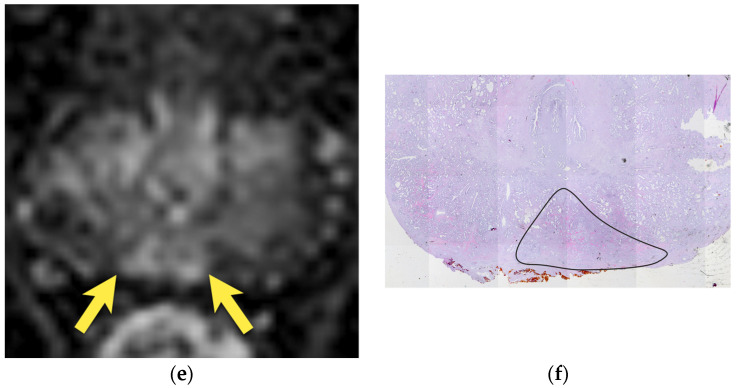
Prostate cancer in the median posterior gland in a 48-year-old patient with PSA 9.75 ng/mL (**a**) axial and coronal (**b**) T2-weighted images show the focal, ill-defined area of low signal intensity (arrows), with marked diffusion restriction on (**c**) DWI b 2000 image (arrows) and (**d**) ADC map (arrows); and early enhancement (arrows) (**e**). The lesion was assessed as PI-RADS 4. (**f**) After radical prostatectomy, PCa GS 4 + 3 was diagnosed in this location (area outlined with black continuous line).

Prostate cancer may also be located in this region, which is sometimes very difficult to differentiate from the image of the central zone. PCa is usually more heterogeneous on T2-weighted images, with ill-defined, fuzzy margins, marked diffusion restriction, and focal, early enhancement [14,15,17] (Figure 6 and Figure 7). The finding of such a lesion should raise suspicion of PCa and require a biopsy.

## 6. Transition Zone—Benign Prostatic Hyperplasia

Benign Prostate Hyperplasia (BPH) affects more than 80% of men and increases with age [26].

BPH is defined as hyperplasia of glandular and fibromuscular stromal cells in the transition zone (TZ) [17] that tends to form nodules of variable sizes and, as a result, causes enlargement of the TZ.

On MRI in T2-w images, benign prostate hyperplasia is depicted as an enlarged TZ of variable signal intensities resembling “organized chaos,” often containing BPH nodules with variable signal intensity. Four types of BPH nodules are distinguished: stromal, glandular, cystic, and mixed [27].

BPH nodules, mainly composed of glandular tissue, are visualized as more hyperintense on T2-w images. In contrast, BPH nodules composed of fibrous stromal tissue and hyperplastic stromal tissue appear as hypointense on the T2-w sequence, sometimes making the differential diagnosis of prostate cancer difficult. In addition, BPH nodules may mildly/moderately restrict diffusion (appear bright on high b values at the DWI sequence and dark on the ADC map) and show rapid enhancement, sometimes with washout on the DCE—features that may resemble cancerous tissue [27,28,29].

**Figure 8:**
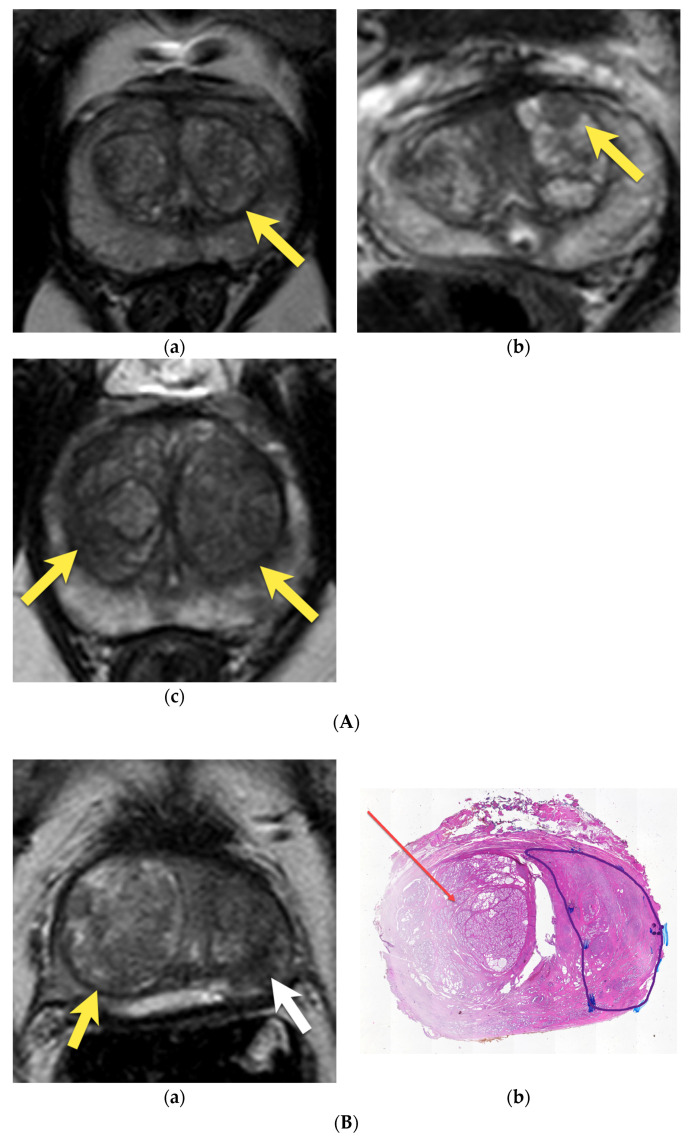
(**A**) BPH nodules on T2W images: (**a**) typical, completely encapsulated BPH nodule (PI-RADS 1) (arrow); (**b**) hypointense, circumscribed stromal nodule within a larger encapsulated nodule (PI-RADS 1) (arrow); (**c**) typical, completely and partially encapsulated BPH nodules (PI-RADS 1 and PI-RADS 2) (arrows). (**B**) A 65-year-old patient with PSA 9.8 ng/mL: (**a**) T2W axial image and (**b**) whole mount histopathology specimen after prostatectomy show the acinar prostate adenocarcinoma in the PZ and TZ of the left lobe of the prostate (white arrow on T2W image (**a**) and outlined and marked area on histopathology specimen after prostatectomy (**b**)) and large completely encapsulated BPH nodule in the right lobe of prostate (yellow arrow on T2W image (**a**) and red arrow on histopathology specimen (**b**)).

**Figure 9:**
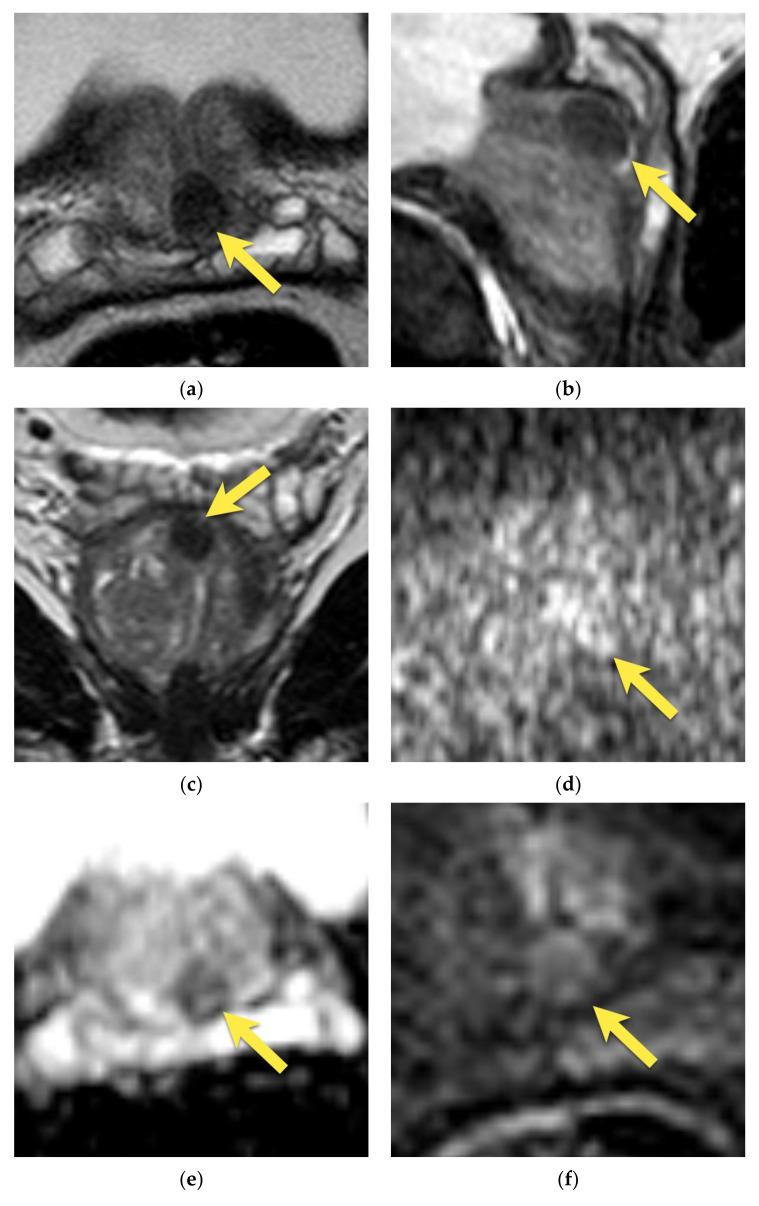

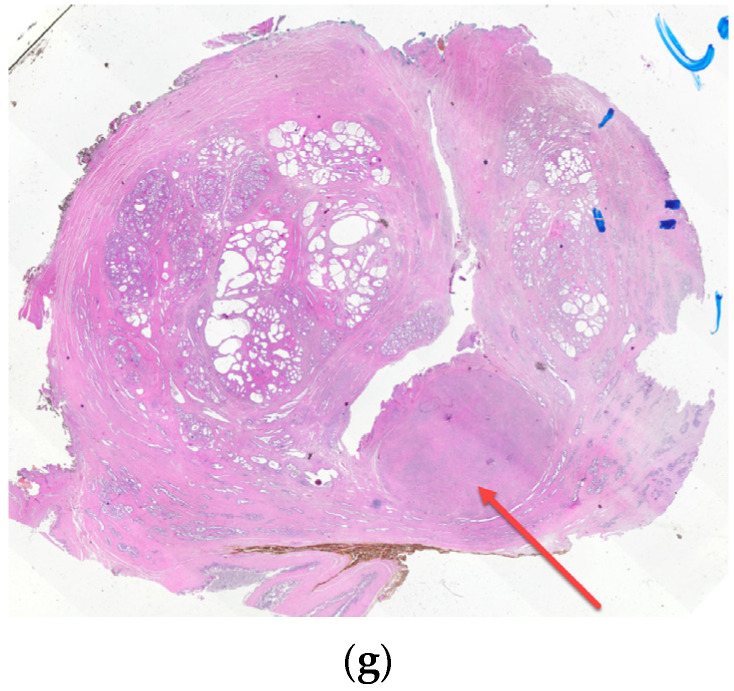
Atypical BPH nodule in the transition zone: axial (**a**), sagittal (**b**), and coronal (**c**) T2-weighted images show homogenous, hypointense, partially encapsulated nodules (arrows), with marked diffusion restriction on (**d**) DWI b 2000 image (arrow) and (**e**) ADC map (arrow); and (**f**) enhancement on DCE (arrow). Overall PI-RADS score for this nodule: PI-RADS 3. (**g**) Histopathologically, it proved to be a stromal BPH nodule (arrow).

According to PIRADS version 2.1, the dominant sequence to assess TZ is T2-w [11]. A typical BHP nodule is round or oval and completely encapsulated, and it should be assessed as PIRADS 1 [11] (Figure 8(Aa,b),B). Partially encapsulated nodules (Figure 8(Ac)) and homogenous, circumscribed nodules without encapsulation or homogenous, mildly hypointense areas between the nodules without diffusion restriction are assessed as PIRADS 2 [11]. Hence, BPH nodules with DWI scores of 4 and 5 are now upgraded from the 2 to 3 PIRADS assessment categories [11] (Figure 9).

**Figure 10:**
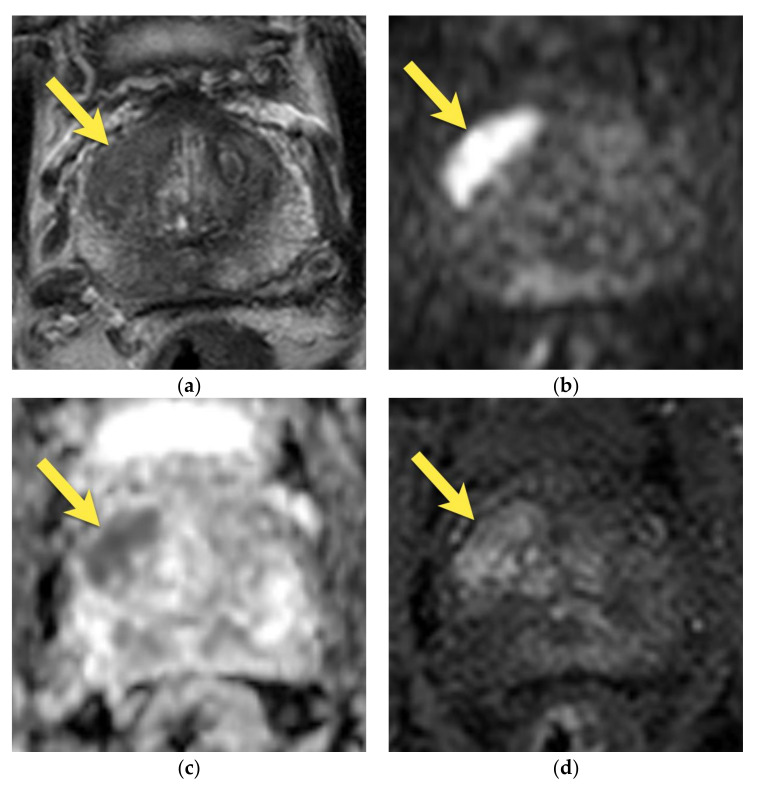

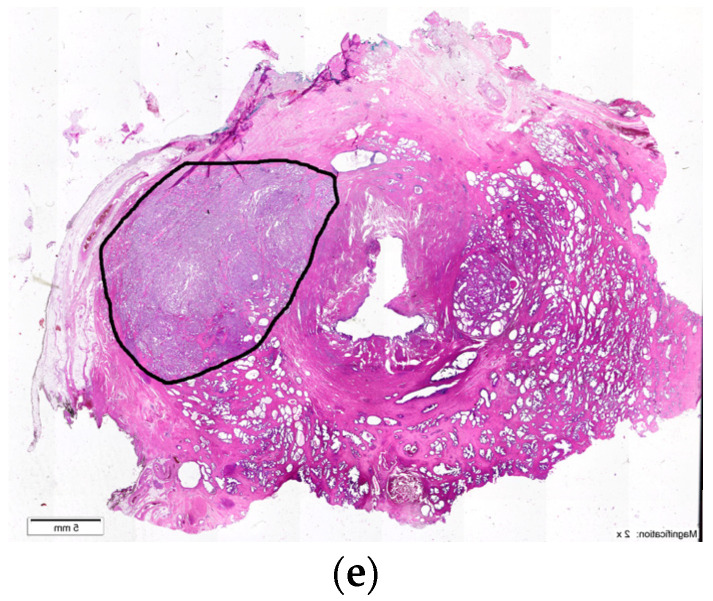
Prostate cancer in the right lobe of prostate, mostly in the transition zone, invading anterior horn of PZ in 74-year-old patient with PSA level 6.84 ng/mL: (**a**) axial T2-w image shows a lenticular, hypointense lesion with “erased charcoal” appearance in the right TZ and anterior horn of PZ (arrow); with marked diffusion restriction (**b**) high signal on DWI b 2000 image (arrow); (**c**) and low signal on ADC map (arrow); (**d**) early enhancement on DCE (arrow); the lesion was assessed as PI-RADS 5; (**e**) whole-mount histopathology after radical prostatectomy revealed prostate cancer GS 3 + 4 in this area (area outlined with black continuous line).

Typical PCa in TZ presents as an ill-defined, lenticular lesion with decreased signal intensity on T2-w images, often compared with an erased charcoal appearance, marked diffusion restriction, and focal, early enhancement that disrupts the “organized chaos” of BPH (Figure 10).

## 7. Ectopic BPH Nodules

**Figure 11:**
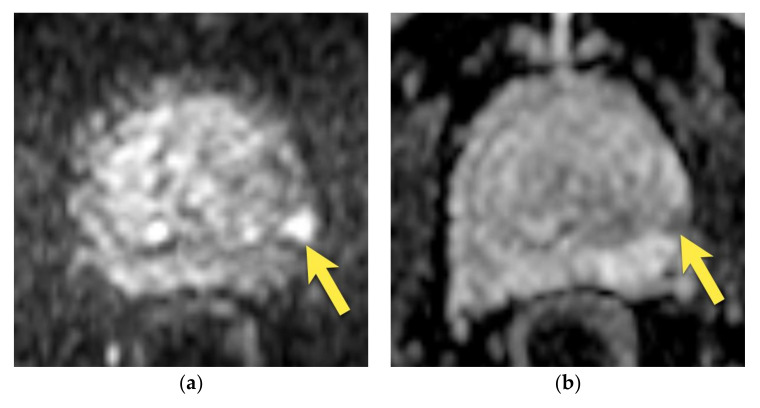

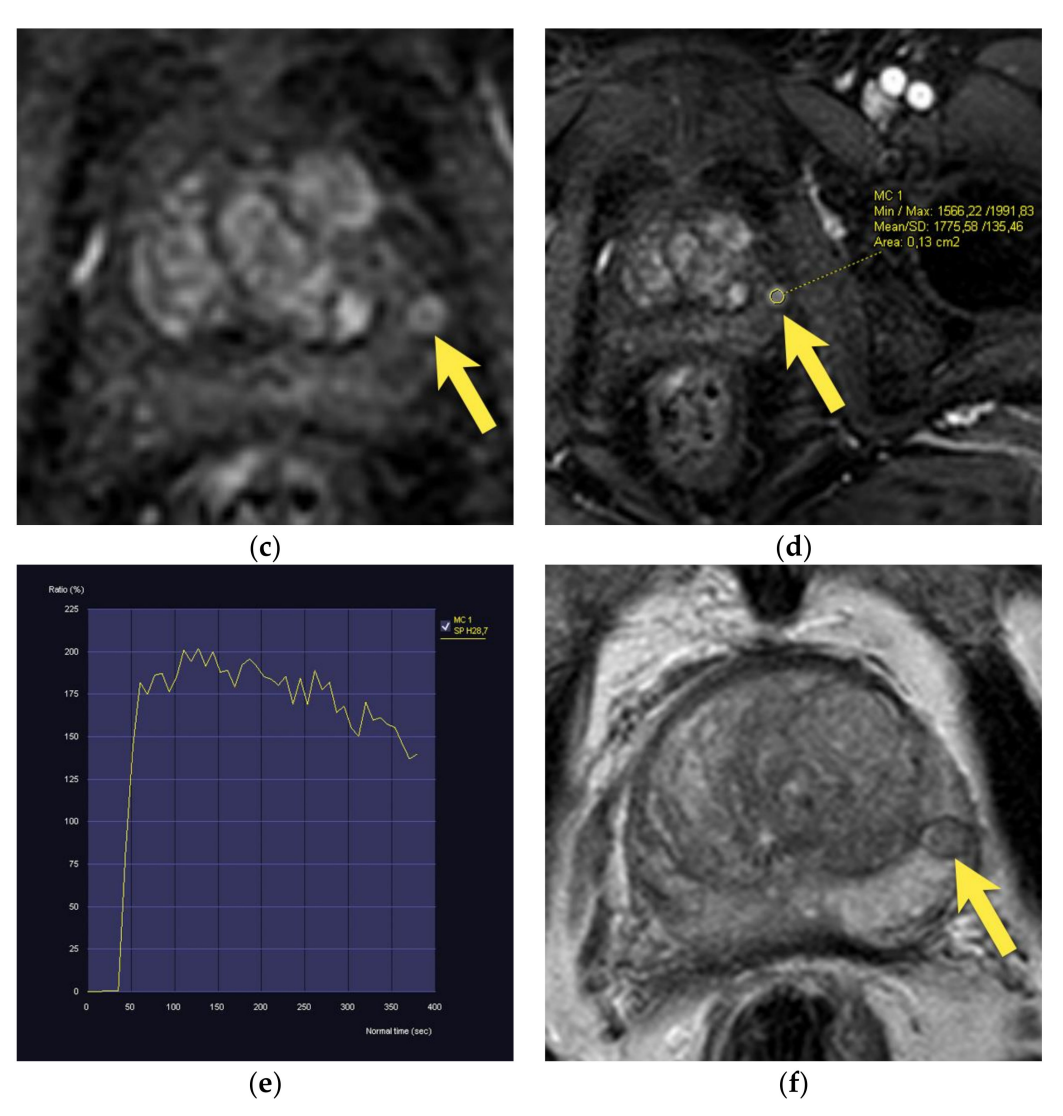
Ectopic BPH nodule in the left peripheral zone: focal lesion with marked diffusion restriction with (**a**) high signal on DWI b 2000 image (arrow); (**b**) low signal on ADC map (arrow); (**c**) early and strong enhancement on DCE; (**d**) ROI for calculation of enhancement curve placed in the lesion on DCE; (**e**) enhancement curve in the lesion shows strong, early enhancement with washout—only in these sequences the lesion appear suspicious for malignancy. However, T2-weighted image (**f**) revealed completely encapsulated BPH nodule, which should be scored as benign—PI-RADS 1.

Ectopically located BPH nodules may simulate cancer, especially when they are located in the peripheral zone. On diffusion-weighted imaging and DCE, they may appear as lesions suspicious of malignancy with marked diffusion restriction and early enhancement [20], sometimes with washout. Despite the DWI, according to PI-RADS ver. 2.1, it is the dominant sequence in the peripheral zone [11]. In this particular case, the key sequence to distinguish them from cancer and make the proper diagnosis is the T2-weighted sequence. A T2-weighted image may reveal well-defined, encapsulated nodules that sometimes contain cysts, morphologically suggesting a BPH nodule (Figure 11).

## 8. Post-Biopsy Hemorrhage

TRUS-guided biopsy remains the gold standard in the detection of prostate cancer in men with elevated PSA levels. However, one of the complications of the biopsy may be hemorrhage, which may lower the diagnostic performance of the MRI.

Post-biopsy hemorrhage resolves over time—hemorrhagic foci are seen in 72.2% of men four weeks after biopsy and decrease to 52% after six weeks [30]. Consequently, a delay of at least six weeks or a more extended period between biopsy and MRI is recommended to decrease hemorrhagic foci, which may hamper the diagnosis or staging of PCa in some cases [11,31].

**Figure 12:**
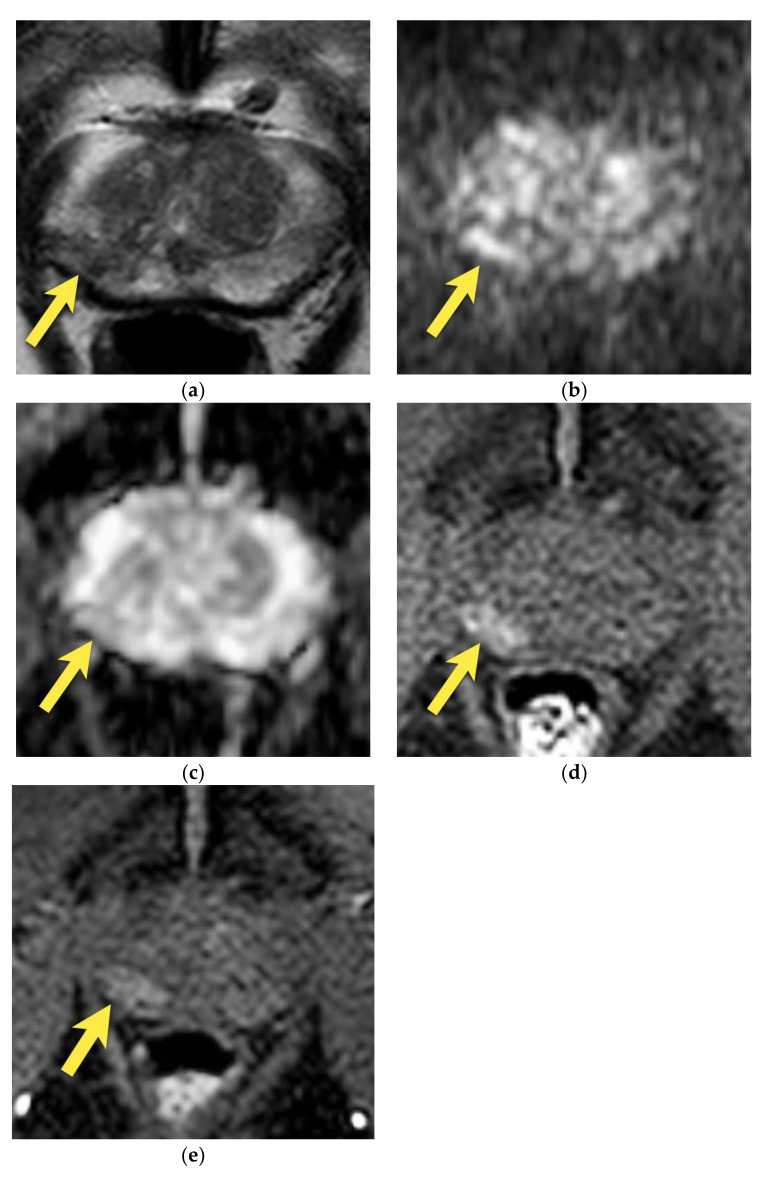
Postbiopsy hemorrhage: (**a**) axial T2-w image shows the area of decreased signal in the right peripheral zone (arrow), with mild diffusion restriction (arrows) (**b**,**c**). However, the native T1-w fat-saturated image reveals the hyperintense signal in this location (arrow), which corresponds to methemoglobin (the product of hemoglobin degradation) after biopsy (**d**). After contrast administration, “pseudo enhancement” is noted (arrow) (**e**). The combination of images is in favor of PI-RADS 2 lesion. The final histopathology did not reveal cancer in this region.

The essential sequence to evaluate hemorrhage is the T1-weighted native sequence without contrast. The hemorrhagic foci (depending on the age of the blood) on MRI may present as hyperintense on T1-w, hypointense on T2-weighted, or hyperintense on T2-weighted. They may restrict diffusion and produce a “pseudo enhancement”, and in this manner, they may mimic PCa (Figure 12). However, assessing the T1 native images usually resolves the uncertainty. Always look at the T1-w native sequence before assessing DCE to avoid confounding the hemorrhage with the enhancement.

**Figure 13:**
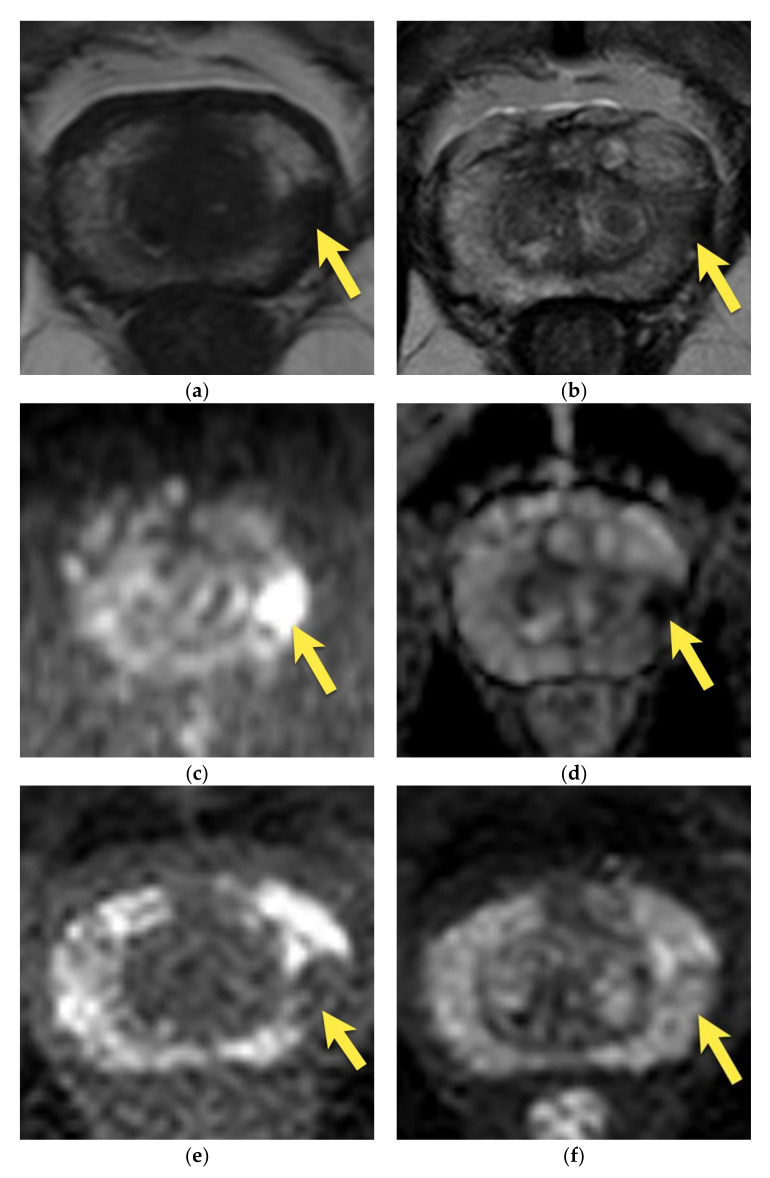

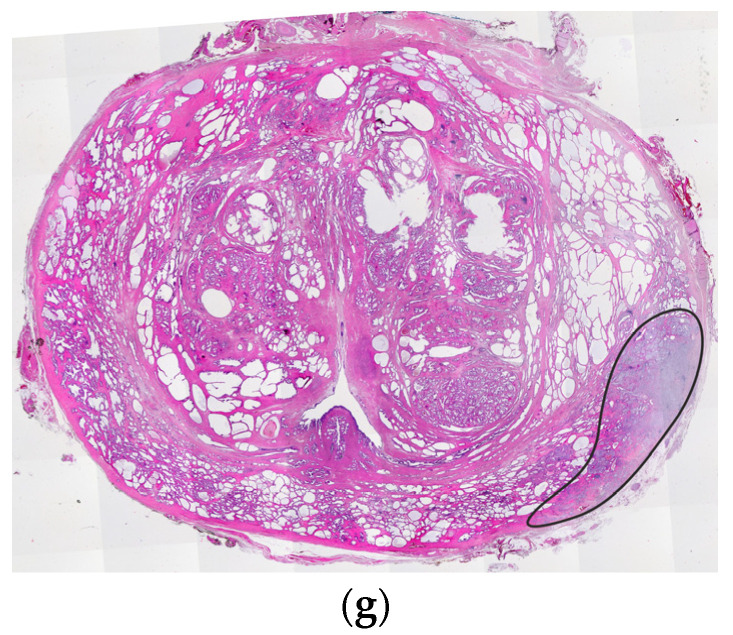
T1 hemorrhage exclusion sign: (**a**) on T1-weighted image, almost the entire PZ demonstrates diffuse hyperintense signal due to hemorrhage, except for a hypointense focus in the left PZ, mid gland (arrow). This hypointense focal lesion in the otherwise hyperintense PZ in T1W images shows: (**b**) hypointense signal on T2W images (arrow) and marked diffusion restriction with (**c**) high signal on DWI b 2000 image (arrow); (**d**) and low signal on ADC map (arrow); (**e**,**f**) and early enhancement after contrast on DCE (arrows). The lesion was reported as PI-RADS 4. (**g**) PCa GS 5 + 4 was diagnosed after radical prostatectomy in the left PZ in this area (area outlined with black continuous line on whole mount histopathology specimen).

On the other hand, the “T1 hemorrhage exclusion sign” phenomenon was described as an area of decreased signal intensity within PZ that is surrounded by an area of high signal on T1-weighted images due to hemorrhage. If such a finding corresponds to a lesion of low signal intensity on T2-w images, which restricts diffusion, it is highly predictive of cancer (95% PPV) [23,32,33] (Figure 13).

The high citrate content in the peripheral zone explains this phenomenon. This metabolite has anticoagulant properties. As a result, a normal prostate peripheral zone may show an increased signal on T1W images due to hemorrhage, usually up to several weeks after the biopsy. Otherwise, prostate cancer shows reduced citrate levels, making it less likely to present prolonged hemorrhagic changes on MRI [32,34].

## 9. Prostatitis

Acute bacterial prostatitis is an acute inflammation of the prostate that causes urinary tract symptoms and pelvic pain [35,36]. The highest incidence of acute prostatitis is reported in patients aged 20–40 and in men over 70 years old [35]. The most common pathogen in acute bacterial inflammation is Escherichia Coli [37].

Chronic prostatitis may be a result of undertreated acute prostatitis, with a lifetime prevalence of 1.8% to 8.2% [16,38].

Prostatitis may occur as focal or diffuse, affecting both the PZ and TZ [16].

It is one of the most common prostate cancer mimics, and it may be impossible to reliably distinguish it from PCa only on MRI images [16], which makes prostate inflammation a major source of false-positive results on MRI [39].

**Figure 14:**
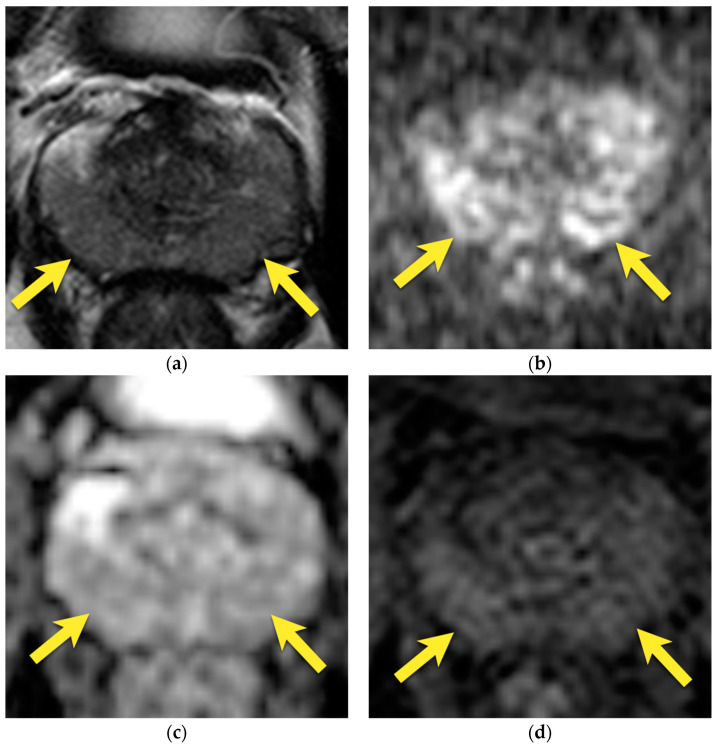
Prostatitis in a 48-year-old patient with elevated PSA 5 ng/mL: (**a**) axial T2-w image shows diffuse, decreased signal in the peripheral zone (arrows), with moderate diffusion restriction; (**b**) diffuse, moderately increased signal on DWI b 2000 image (arrows); (**c**) diffuse, moderately decreased signal on ADC map (arrows); (**d**) mild, diffuse enhancement of PZ (arrows). Findings reported as PI-RADS 2.

On MRI, diffuse prostate inflammation presents as an area of decreased signal intensity on T2-weighted images, with usually mild to moderate diffusion restriction and increased perfusion on DCE [11,16] (Figure 14). However, in contrast to cancer, prostatitis usually appears as a more diffuse, lobar, or linear area with indistinct borders rather than a focal, rounded, or oval lesion [15]. In addition, the signal changes on T2-weighted images and DWI are usually mild to moderate and slightly less pronounced than in cancer [11]. Therefore, the patient’s clinical history and follow-up after treatment are crucial in making the proper diagnosis [14,16].

**Figure 15:**
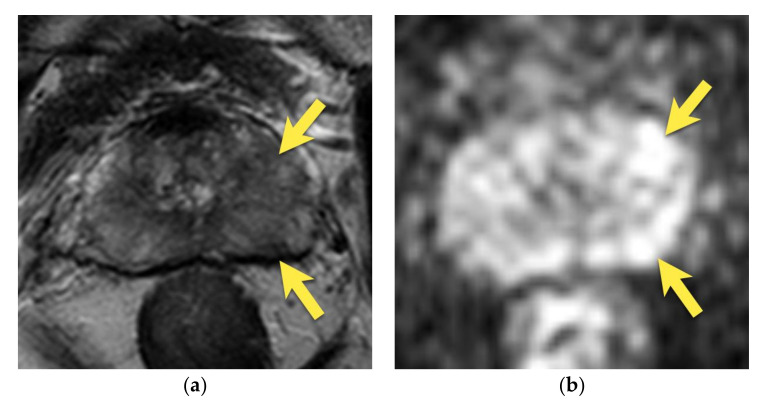

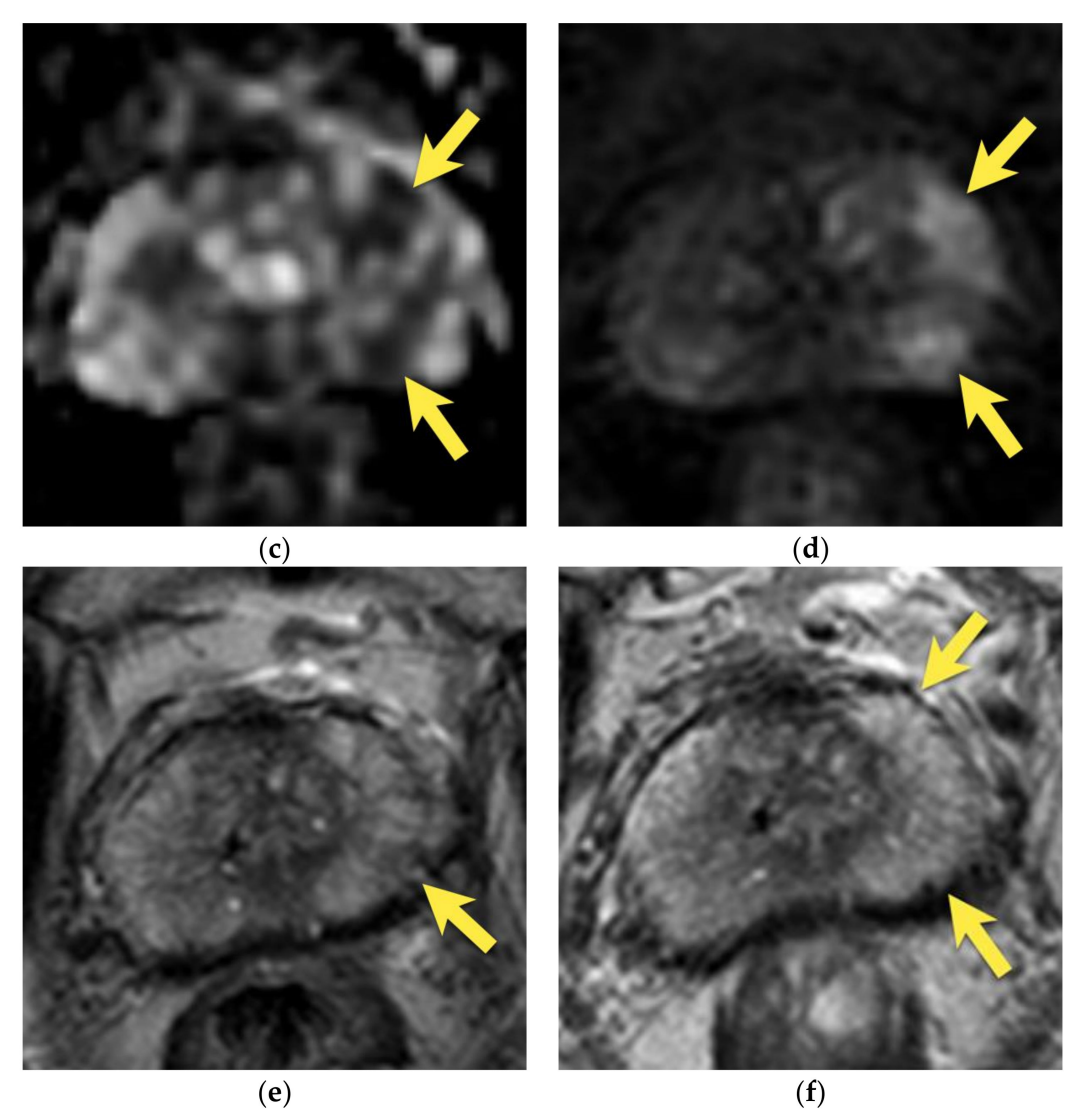
Patient 82 years old, with PSA 7.49 ng/mL: (**a**) axial T2-w image shows diffuse, moderately decreased signal in the peripheral zone with two foci of lower signal intensity (arrows), which demonstrate focal, marked diffusion restriction (arrows) (**b**,**c**) and early enhancement on DCE (arrows) (**d**). The lesions were classified as PI-RADS 4 and biopsied. A histopathological examination did not reveal any cancer. Follow-up MRI at five months showed significant resolution of the lesions (arrow) (**e**) and, after one year, complete disappearance of the lesions (arrows) (**f**). PSA decreased to 0.3 ng/mL. Images demonstrate lesions in the course of prostatitis.

**Figure 16:**
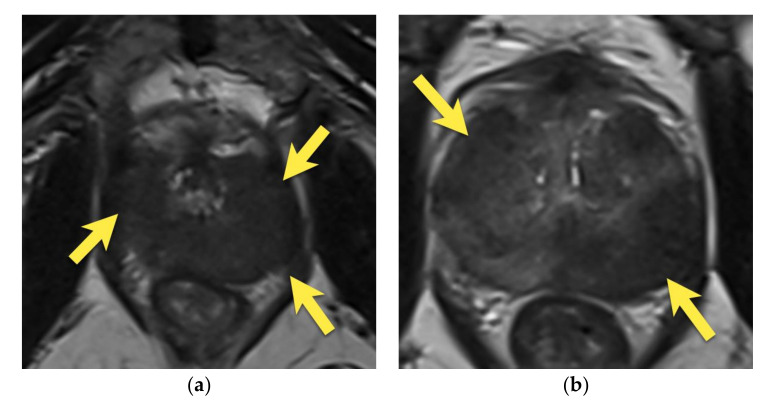

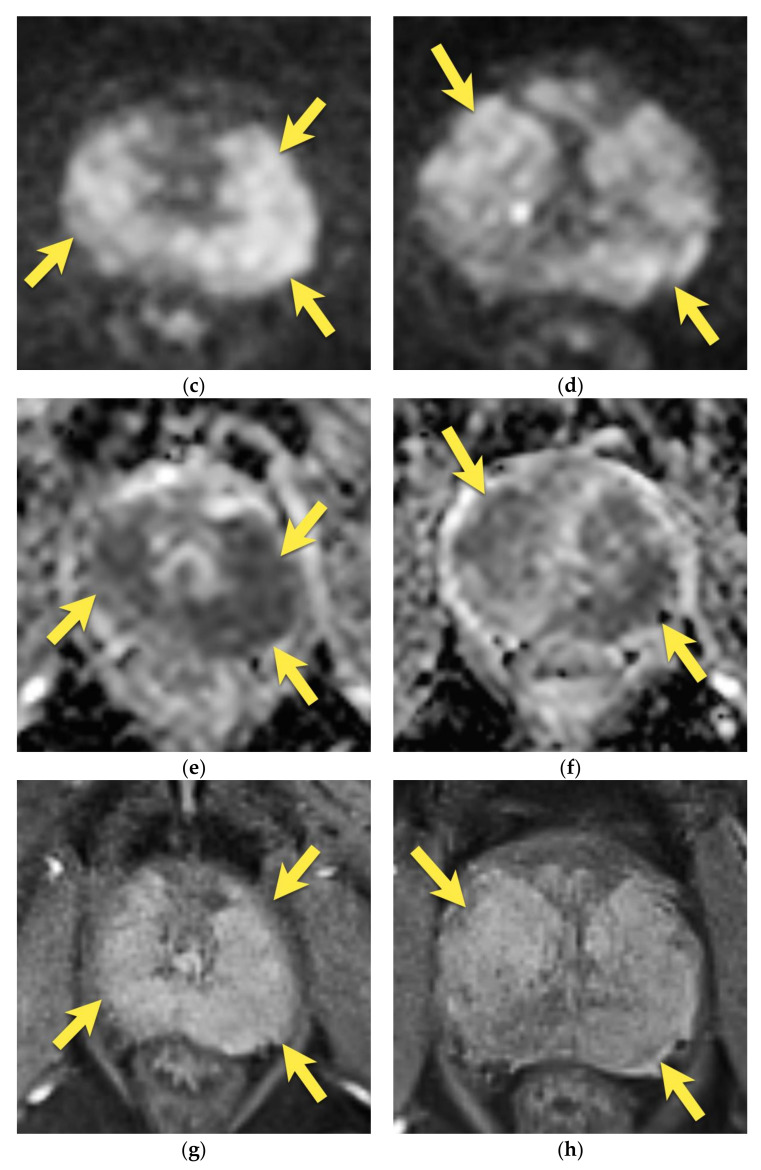
Patient 76 years old, with PSA 10 ng/mL: (**a**,**b**) axial T2-w images show diffusely decreased signal in the entire prostate, with large, diffuse areas of lower “erased charcoal” signal intensity, which include both peripheral and transition zones in the apex and mid gland, more pronounced on the left, with bulging of the capsule in the apex on the left (arrows), marked diffusion restriction (arrows) (**c**–**f**), and strong, early enhancement on DCE (arrows) (**g**,**h**). The lesions were classified as PI-RADS 5 and biopsied. A histopathological examination revealed confluent foci of intensive active and chronic inflammation with glandular destruction and focal granuloma formation. No fungal infection or cancer was revealed. After treatment, PSA decreased to 4.6 ng/mL. The images listed above demonstrate diffuse, active granulomatous prostate inflammation.

Focal prostatitis and granulomatous prostatitis may be indistinguishable from prostate cancer both on MRI, where they present as a focal lesion with low T2-signal, marked diffusion restriction, and early enhancement, and on clinical examination, where they present as a focal or diffuse area of stiffness of the gland and a raised PSA level [16]. According to PI-RADS ver. 2.1, the MRI lesions described above could be assessed as PI-RADS 4 or 5 [14]. Therefore, histopathological verification of such lesions is necessary to make an accurate diagnosis and exclude the presence of a neoplastic tumor [14] (Figure 15 and Figure 16).

**Figure 17:**
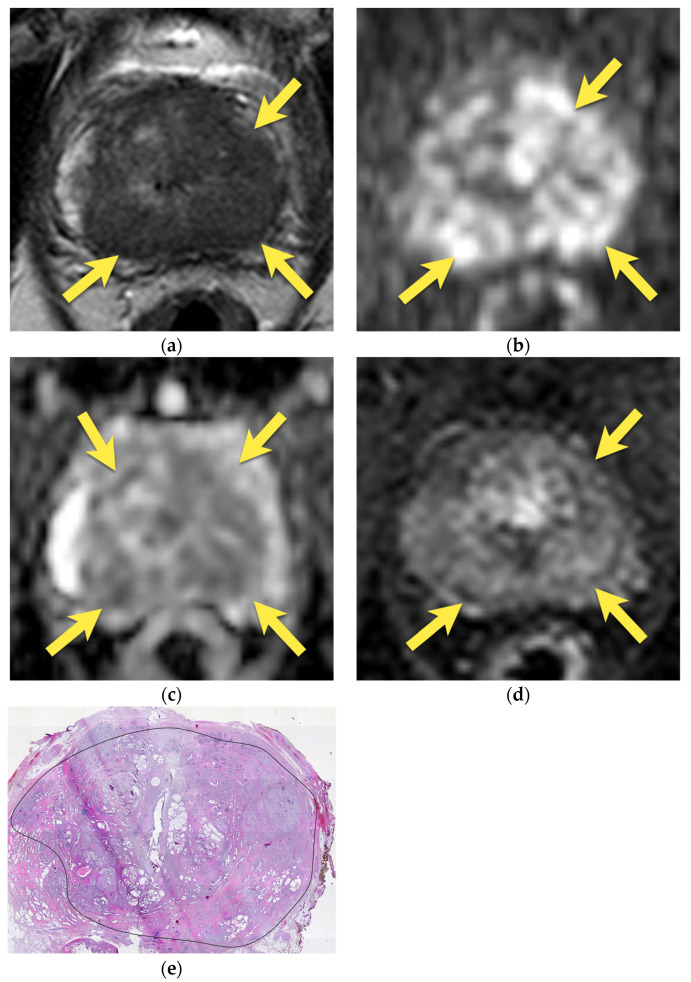
Patient 61 years old, PSA 5 ng/mL. (**a**) Axial T2-w image shows a diffuse low intensity signal of “erased charcoal” appearance in almost entire prostate gland, involving peripheral and transition zones (arrows); with marked diffusion restriction (**b**) high signal on DWI b 2000 image in almost entire prostate (arrows); (**c**) corresponding to low signal on ADC map (arrows); (**d**) and early enhancement on DCE (arrows); The lesion was assessed as PI-RADS 5. (**e**) diffuse prostate cancer GS 5 + 4 was revealed in this area on whole mount histopathology specimen after radical prostatectomy (area outlined with black continuous line).

For comparison, the diffuse infiltration of prostate cancer in almost the entire prostate gland, with the low signal intensity of an “erased charcoal” appearance, strong diffusion restriction, and early enhancement, is depicted in Figure 17.

## 10. Prostatic Abscess

**Figure 18:**
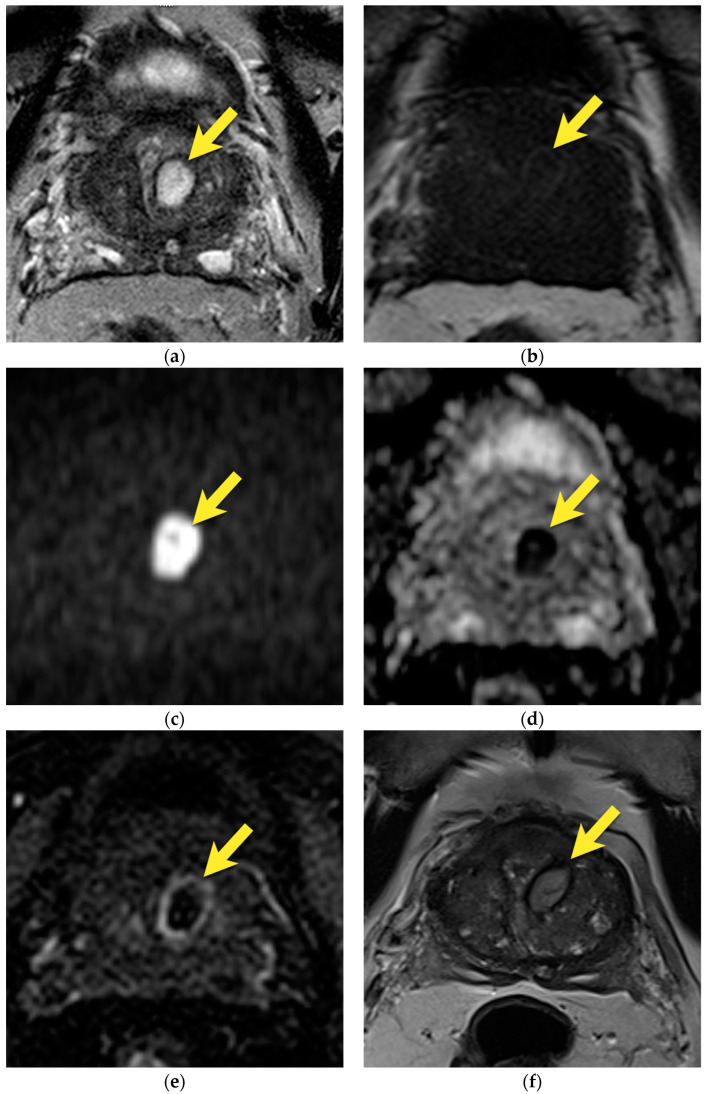
Abscess in the transition zone of the prostate: (**a**) axial T2-w image shows hyperintense, oval lesion in the left transition zone at the base of the prostate, with hypointense rim (arrow); (**b**) axial T1-w image shows isointense oval lesion, with slightly hyperintense rim (arrow); (**c**) with marked diffusion restriction high signal on DWI b 2000 (arrow); (**d**) corresponding to very low signal on ADC map (arrows); (**e**) rim enhancement on DCE (arrow); lesion was reported as PI-RADS 2; (**f**) after antibiotic treatment, the abscess persisted, but in T2-w images, its signal changed to intermediate intensity (arrow).

Prostatic abscess most commonly develops as a complication of acute prostatitis, biopsy, or other prostate procedures such as cryotherapy, brachytherapy, and intravesical BCG therapy [40]. In addition, it frequently affects men with diabetes or immunodeficiency disorders [40]. On MRI, it is visualized as a lesion with increased signal in T2-w images and iso–low signal in T1-w images, in the center showing strong diffusion restriction, a very low ADC value, and ring-type enhancement on DCE of the pseudocapsule on the periphery [41] (Figure 18). In some cases, it may show an iso to slightly lower signal in T2-w images [14], which, together with the strong diffusion restriction, may be misleading. However, a very low ADC, ring-type enhancement, and usually high T2-w signal with a correlation with clinical data (e.g., fever, previous urological instrumentation) guide to a correct diagnosis.

## 11. Quality of Images

Image quality, along with a radiologist’s expertise, plays a crucial role in an accurate diagnosis. Images of poor quality, motion artifacts, rectal gas, and hip prostheses may lead to uncertainty or false positive or negative results, even when assessed by experienced radiologists. The Prostate Imaging Quality (PI-QUAL) score was invented to evaluate the diagnostic quality of images from mpMRI [42]. Firstly, obtaining high-resolution T2-w images, high-quality diffusion-weighted imaging, and DCE is highly recommended according to PIRADS v. 2.1 guidelines [11,43]. Secondly, artifacts due to bowel motion and distention of the rectum should be eliminated—it has been reported that referral of a rectal enema and a special diet before, as well as administering anti-spasmodic drugs during the examination, should diminish these artifacts and improve the quality of the images [44,45,46].

**Figure 19:**
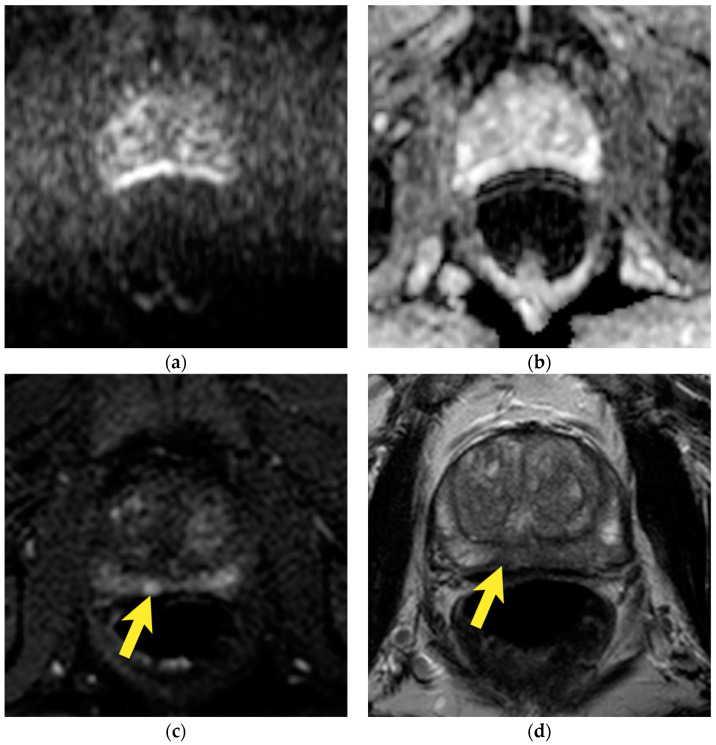

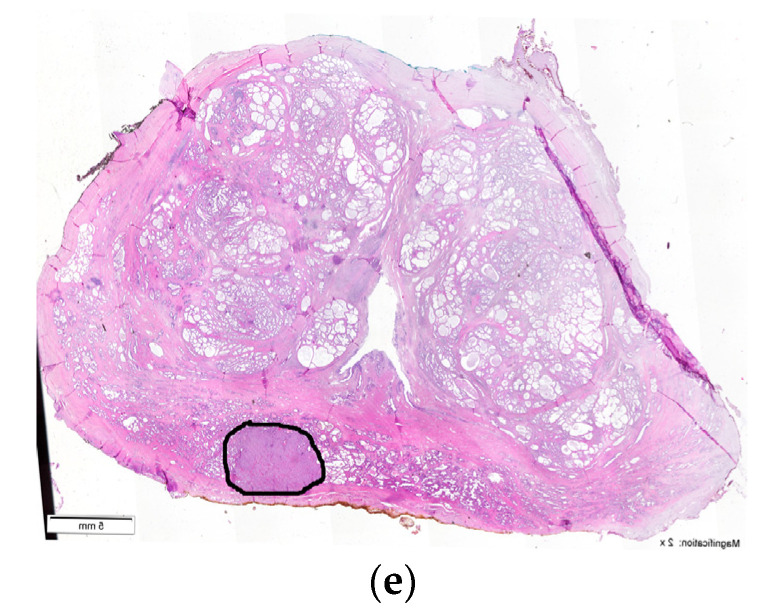
DWI sequence affected by artifacts from the gas in the rectum—rendering interpretation of the PZ in posterior gland impossible: (**a**) DWI b 2000; (**b**) ADC map. However, in this patient, a focal early enhancement is visible on DCE (arrow) (**c**), which corresponds to a poorly visible, hypointense focal lesion on T2-weigted image (arrow) (**d**), on the background of a larger area of reduced signal intensity; the lesion was reported as PI-RADS 4; (**e**) whole-mount histopathology after radical prostatectomy reveals prostate cancer GS 4 + 3 in this location in the right PZ (area outlined with black continuous line).

A good signal-to-noise ratio should also be maintained [43]. DWI, which plays a crucial role in the detection of PCa in the peripheral zone, is the most susceptible to artifacts (Figure 19). Recent MR acquisition techniques, e.g., parallel imaging and motion reduction techniques, as well as right-left phase encoding instead of anterior-posterior, may reduce those artifacts [27,43].

## 12. Conclusions

Recently, mpMRI has evolved as a pivotal diagnostic imaging modality to detect or rule out significant prostate cancer. However, despite the high accuracy of mpMRI in detecting and staging PCa, false positive and false negative results are reported, and potential pitfalls in interpretation exist. Therefore, radiologists reporting prostate mpMRI should be aware of those pitfalls to improve their diagnostic accuracy.

## Figures and Tables

**Table 1:** Summary of prostate cancer mimics in relation to the anatomic location [14,15,16,17].

	Type	Location
Anatomic structures	Anterior fibromuscular stroma	Anterior prostate
	Central zone	Base of the prostate and posterior middle peripheral zone
	Periprostatic venous plexus	Peripheral zone
Benign pathology	Stromal BPH nodule	Transition zone and occasionally the peripheral zone (as ectopic BPH nodule)
	Ectopic BPH nodule	Peripheral zone
	Diffuse and focal prostatitis	Peripheral zone is more often involved, adjacent transition zone may also be affected
	Abscess	Peripheral or transition zone
	Hemorrhage	Peripheral or transition zone

## Data Availability

The detailed data presented in this study are available from the corresponding author upon request.

## Abbreviations

mpMRI—multiparametric MRI; PCa—prostate cancer; GS—Gleason score; PSA—prostate-specific antigen; DWI—diffusion-weighted imaging; ADC—apparent diffusion coefficient; DCE—dynamic contrast enhanced; PZ—peripheral zone; TZ—transition zone; AFMS—anterior fibromuscular stroma; PI-RADS—Prostate Imaging—Reporting and Data System; ROI—region of interest
